# Scite

**DOI:** 10.5195/jmla.2021.1331

**Published:** 2021-10-01

**Authors:** Stacy Brody

**Affiliations:** 1 sbrody98@gwu.edu, Reference and Instruction Librarian, Himmelfarb Health Sciences Library, George Washington University, Washington, DC

## Abstract

**Scite.** Scite Inc., 334 Leonard St., Brooklyn, NY 11211; https://scite.ai/; tiered pricing model with free, basic ($7.99/month), premium ($19.99/month or $100/year), premium+ ($59.99/month), and enterprise plans.

Scite (https://scite.ai/) was founded by Josh Nicholson and Yuri Lazebnik and previously funded by the National Science Foundation (NSF) and National Institute on Drug Abuse (NIDA) [1, 2, 3]. The Scite database contains over 800 million citation statements [4] tagged by a machine learning algorithm as supporting, mentioning, or contrasting the findings of cited articles [5] and by their locations in the citing articles (introduction, results, methods, discussion, or other). Scite also provides a count of editorial notices for each article. Users can search the website and install plug-ins for browsers Chrome and Firefox and reference management tools such as Zotero. Additional tools include reports and dashboards, badges, and automated reference checks. Scite can be used by researchers to locate evidence and evaluate references; librarians to enhance research impact projects; publishers and editors to check reference lists of submissions [6, 7]; and journals, publishers, and databases to create context and showcase impact [4, 8, 9].

## SCITE AIMS TO CONTEXTUALIZE THE CITATION COUNT

Citation counts are commonly used to determine the impact of articles or bodies of work. Academic search engines might use citation counts in relevancy ranking algorithms [10]. In reference consultations and library instruction sessions, I have learned that students use citation counts to select articles. Faculty and researchers are evaluated on their scholarly works, and citation counts are often included in tenure packets.

Citation count is a unidimensional metric. Each citation statement, no matter where it appears in the citing article or whether it provides supporting or contrasting evidence, counts equally as 1.

A highly cited article may be a seminal paper cited by all related articles in the introduction to be taken seriously by reviewers. Perhaps the highly cited article clearly describes an established method or best practices and is commonly mentioned in methods sections (e.g., [11]). Alternatively, an article may be cited often because the results are repeatedly being overturned [12]. The use and citation of disputed articles can impact research trends, clinical practice, and patient health and well-being [13, 7]. It is important to note here that, because “(a) scite classifies citation statements at the level of the claim, not the full paper, and (b) the citing article making the contradicting claim itself could be without merit,” “a contradicting citation statement does not necessarily mean the cited paper is wrong” [14].

Ultimately, citation count alone does not show how or why an article is being referenced. Yet, this count is used to determine the impact of scholarship (i.e., [15]). The plethora of LibGuides on research impact and citation counts, among other metrics, suggests librarians are highly engaged in this space, that faculty seek new ways to demonstrate their impact, and that administrators and funders are keenly interested in tools to evaluate faculty and funded researchers. We and our researchers could benefit from using tools like Scite to contextualize these counts.

## SCITE USES TEXT MINING AND ARTIFICIAL INTELLIGENCE

To classify citation statements as supporting, mentioning, or contrasting, Scite uses a proprietary machine learning model trained on over 40,000 citation statements.

Scite continues to build partnerships with publishers to gain access to full-text articles to allow for the use of text mining. Scite signed an agreement with Sage in early 2021 [16], adding to preexisting indexing agreements with publishers from BMJ to Wiley [4].

## SCITE TOOLS FOR RESEARCHERS

Researchers use Scite to see how others cite their publications and how their results fit into the larger landscape. Are their works being referenced in methods sections? Are their results being supported or contrasted in new works? Researchers can display this information by customizing and adding the Scite badge to their websites.

With a free account, researchers can create a very limited number of reports and visualizations and set up author alerts. A paid account allows researchers to access the reference check feature, which alerts authors of the uploaded manuscript to references that may have been disputed, retracted, or otherwise received an editorial notice. Most publishers do not check reference lists for retracted or disputed papers; authors can ensure they are citing articles appropriately by using the Scite.ai tool to alert them to references that may be in dispute [7].

## CITE RESPONSIBLY

Using the Scite browser and Zotero plug-ins, librarians, researchers, and students can see more nuanced citation counts, which can help them better evaluate articles. For instance, a student who sees a high citation count might opt to reference an article. However, if the student notes a high number of contrasting citation statements, the student might be encouraged to select a different piece, the findings of which have not been in such dispute.

**Figure 1:**
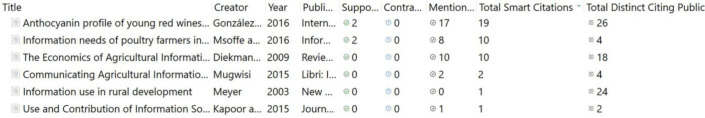
In the desktop version of Zotero, the Scite plug-in displays columns with counts of supporting, contrasting, and mentioning citation statements.

Scite smart citations are beginning to appear on databases, including EuropePMC, and journal websites [4, 9]. Smart citations add one more way to evaluate articles in the broader context of research and scholarly communication. Librarians should keep up with these developments to prepare for questions from their research communities.

Publications posted on nonpublisher websites or stored in personal reference libraries may lack updated information and context, for example, links to errata or retraction notices [17]. The Scite browser plug-in provides context and insights across databases and platforms, so long as the underlying metadata is present. The Scite Zotero plug-in offers access to the Scite data, embedded in a reference library and regularly updated with information on citation statements to saved articles. This can help users quickly check reference libraries for papers, the findings of which have been contrasted.

## SUPPORT SCHOLARLY IMPACT WORK

A Scite dashboard provides citation counts, with the nuance of supporting, mentioning, and contrasting citations, as well as counts of editorial notices related to a set of papers. Dashboard creators can set notifications for events such as new citations to papers within the collection or search results set. Additionally, the dashboard calculates the Scite index for two-year, five-year, and lifetime citations. The Scite index is calculated by dividing the number of supporting citation statements by the sum of supporting and contrasting citation statements [18]. As in all impact metrics work, papers must be old enough to have accumulated cites of a significant amount. Scite also offers a searchable table of Scite index values for journals in their system [19].

## SCITE CAN ALSO BE USED TO CONDUCT RESEARCH

The Scite corpus has been used to check the quality of references cited in Wikipedia articles [14]. Scite data could be used in reviews looking at how datasets, software, and research tools are used by restricting a citation search to publications citing tools in their methods sections [20]. While some databases, for instance PubMed Central, offer the ability to restrict searches to the materials and methods sections of papers, this feature is not widely available. The use of Scite to locate studies employing various tools and techniques as part of their methodology could be a unique and valuable use of the database.

Another potential avenue of research could involve the use of the reference check feature, which looks at evidence syntheses, for example, systematic reviews and clinical guidelines, for the inclusion of references with significant numbers of contrasting statements from other publications or editorial concerns.

## USE SCITE WITH A GRAIN OF SALT

The algorithm which classifies citation statements as supporting, mentioning, or contrasting is still evolving. To reflect this, Scite displays confidence levels for classifications and offers opportunities to flag misclassified cites [21]. Currently, “ ‘Most citations [in Scite] are classified as “mentions”, because the classifier is trained to be cautious …' says [Giovanni] Colavizza [an AI scientist at the University of Amsterdam and visiting researcher at the Alan Turing Institute in London], who is a user of the platform and whose team has analyzed data from the start-up [Scite] in the past” [22]. A high count of mentioning citation statements may provide little more nuance or detail than a total citation count.

As with all citation indexes, methods and data sources vary. While Scite continues to partner with publishers to expand the database, the corpus to which they have access is incomplete. In Scite, the count is the number of individually extracted citation statements, not the number of citing articles. Due to this variation, as well as the corpus from which the statements are extracted, different numbers are found across platforms like Scopus, Web of Science, PubMed, Google Scholar, and Dimensions, among others. By way of example, the article, “PRESS peer review of electronic search strategies: 2015 guideline statement” [23], has been cited by 493 articles in PubMed, 835 publications with 590 extracted citation statements in Scite, 740 publications in Scopus, 671 publications in Web of Science, and 1,078, according to Google Scholar (as of June 4, 2021).

As Scite's founders note, “Our results should be considered with caution given the limitations of the model precision, the current limited coverage of articles analyzed by scite, and the fact that articles that could not be linked to a DOI in the data set were excluded” [14].

## SCITE ALTERNATIVES

While Scite is imperfect, the algorithm, corpus, and accompanying tools continue to evolve to provide nuanced insights into how and where works are being used. Scite fits into a larger landscape of tools to contextualize research articles and impact. While Scite restricts its focus to publication types with DOIs, mainly research articles and scholarly publications, Altmetrics, for instance, expands the scope to social media and blogs, reference management tools like Mendeley, policy documents, and other publication types.

As of April 2021, Edifix, a reference editing tool, “identifies retracted or corrected articles and articles published in journals with fraudulent or unprofessional practices,” as determined by Cabells Predatory Reports [24]. In contrast to Scite, the Cabells check provided by Edifix relies on journal-level, rather than article-level, data.

CrossRef's Cited By service works with publishers to create and maintain metadata-enabled connections among citing and cited articles [25]. This program enables linking and updates to citation counts but does not provide the same nuance as Scite does in terms of where the citation statement is located in the citing paper and whether the citation statement is supporting, contrasting, or mentioning the cited article.

Depending on the features of interest, free and low-cost alternatives to Scite are available. If the most important feature is the identification of retracted papers, the Retraction Watch plug-in for Zotero flags papers that have been retracted and identified as such by Retraction Watch [26].

## PRICING OPTIONS

Scite offers five pricing tiers, including a free option. With a free account, users can search the smart citation database, generate one Scite report and one visualization per month, create one custom dashboard, and set author and paper alerts. Up to 1,000 search results can be exported as comma-separated values (CSV) files, which include bibliographic metadata and unique identifiers; supporting, contrasting, mentioning, and total cites; and links for the full Scite reports. As described above, a dashboard provides data on smart citations for a set of articles. A Scite report provides detailed results, including each extracted citation statement and its classification as supporting, mentioning, or contrasting, for an individual article. The free account does not include the ability to export Scite reports. Due to the one per month report limit and the restriction on report downloads, real and regular use is severely restrained under a free account, though alerts can be useful for current awareness and monitoring.

A basic plan, costing $7.99 per month, offers unlimited reports and visualizations, plus the option to export reports as CSV files, a valuable feature for librarians supporting research impact and bibliometrics projects.

The premium plan, at $19.99 per month, adds unlimited custom dashboards with up to 1,000 DOIs, unlimited reference checks, unlimited alerts, and unlimited saved search alerts with up to 1,000 DOIs. With higher limits, librarians or principal investigators can monitor and regularly report the impact of article sets, such as those generated by larger departments. Researchers can use the reference check tool to ensure they are not citing articles that have been highly disputed in their reference lists.

The premium+ plan, at $59.99 per month, offers the additional features of citation statement searching and alerts.

Enterprise plans provide higher article limits, access to data and functionalities, such as APIs, and additional support.

## SUMMARY

As the technology, tools, and articles available for text mining continue to evolve, the full value of Scite remains to be seen. Use of the free browser and reference manager plug-ins can provide some interesting insights to reference librarians, students, and researchers. Any purposeful uses require paid subscriptions.
